# Loperamide Overdose

**DOI:** 10.7759/cureus.4753

**Published:** 2019-05-25

**Authors:** Casey Arnold, Carmen J Martinez Martinez

**Affiliations:** 1 Emergency Medicine, Advent Health Florida Hospital, Orlando, USA

**Keywords:** loperamide overdose, imodium® overdose, opioid, long qt syndrome, torsades

## Abstract

In light of the opioid epidemic, cardiac complications following a loperamide overdose are a growing concern for patients presenting to the emergency department (ED). Here, we present the case of a 35-year-old male with long QT syndrome who presented following a loperamide overdose and was receiving multiple shocks because of the resulting arrhythmias and electrolyte disturbances. It is necessary for emergency physicians to be aware of loperamide overdoses because cases have been increasing over the past several years and the cardiac complications can be life-threatening.

## Introduction

Loperamide (Imodium®) is an over-the-counter anti-diarrheal medication abused by some patients to either achieve opioid-like euphoria or self-medicate the symptoms of opioid withdrawal [1]. The use of this drug of abuse is on the rise with the number of cases of reported overdose increasing each year [2]. Unfortunately, patients must use doses much higher than recommended to achieve systemic opioid effects with loperamide. These overdoses result in cardiac arrhythmias and electrolyte disturbances that can be fatal [1,3-4]. Due to the increasingly problematic opioid epidemic, the emergency physician needs to be aware of loperamide overdose as a possible cause for arrhythmia in the toxicology patient. In this case, we present a 35-year-old male with a loperamide overdose, leading to multiple shocks from his automated implantable cardioverter-defibrillator (AICD) because of the resulting arrhythmias.

## Case presentation

A 35-year-old male with a history of AICD placement for long QT syndrome by a local cardiologist six months ago presented to the ED by ambulance for altered mental status and frequent shocks from AICD secondary to a loperamide overdose. He was unable to provide a history of more than “yes" or "no” because of his agitation, so the most history was obtained from emergency medical services (EMS), with confirmation by the patient. EMS stated that the patient was having dinner with his spouse when the spouse noticed that he had a brief loss of consciousness for several seconds. The spouse stated that when the patient awoke, he became very anxious, diaphoretic, and irritable; it was unclear if the defibrillator had fired at that time. On arrival, EMS gave the patient 2 mg of naloxone IV without any effect. They stated that en route to the facility, they noted that the AICD had fired several times, causing the patient much anxiety and pain. EMS also stated that they found a bag full of loperamide tablets inside a purse-string bag near the patient, which they brought into the department for identification. They stated that the patient had a history of IV drug abuse and had overdosed on loperamide in the past when he had been unable to obtain opiate medications. In the ED, the patient was anxious and diaphoretic and was shocked by his defibrillator every three to four minutes for runs of polymorphic ventricular tachycardia (Torsades). A review of symptoms was negative, except for tachycardia, anxiety, and diaphoresis. Initial vital signs were a heart rate of 120, respiratory rate of 24, oxygen saturation 99% on room air, and blood pressure 138/64. Physical exam showed that the patient could not hold an extended conversation; he was anxious, had reactive mydriasis, and an AICD in place in the left chest, without signs of infection; otherwise, the physical was normal.

**Figure 1:**
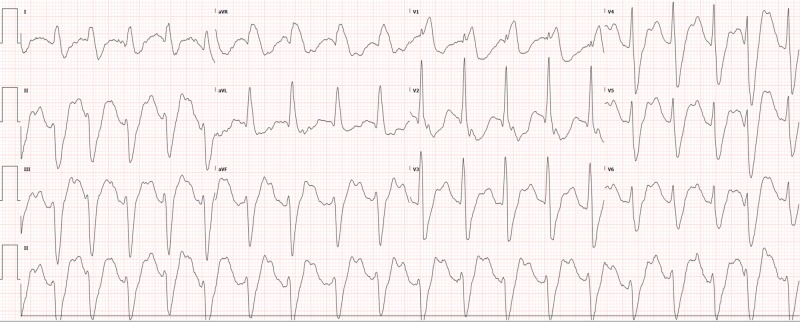
Initial ECG in the resuscitation room upon arrival ECG: electrocardiogram

**Figure 2:**
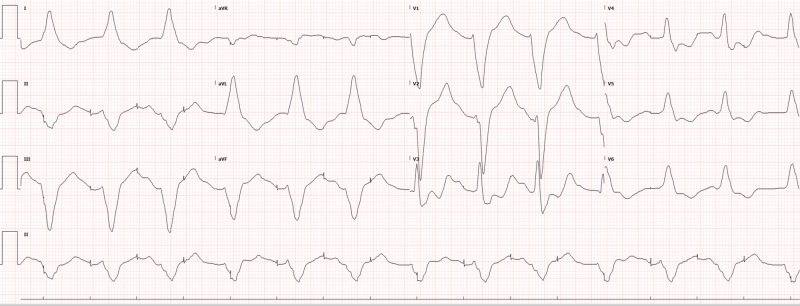
ECG after 5.5 hours of antiarrhythmics (lidocaine) and electrolyte replacement showing a failure to sense and capture ECG: electrocardiogram

**Figure 3:**
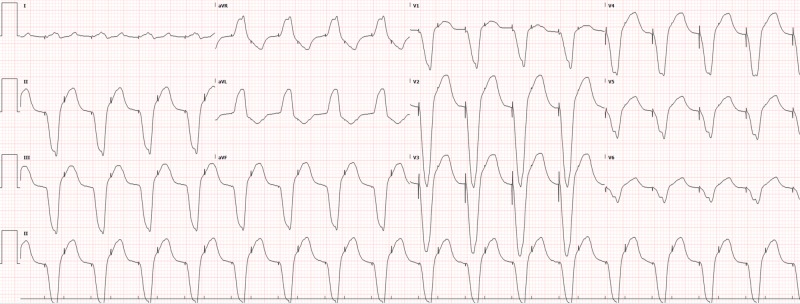
ECG after two days, showing AV dual paced complexes ECG: electrocardiogram; AV: atrioventricular

**Figure 4:**
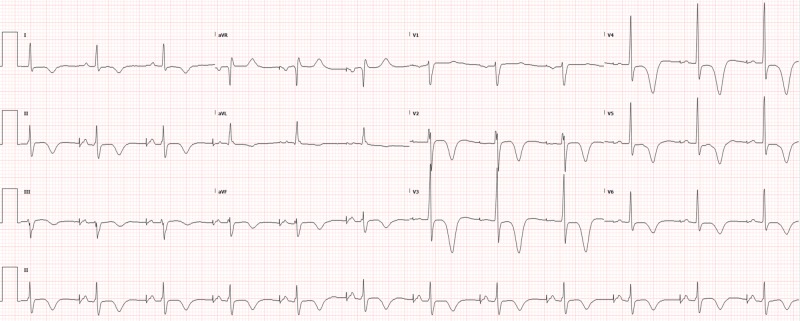
ECG 10 days later, when the patient presented back to the ED for routine chest pain workup, showing his baseline atrial paced rhythm restored ECG: electrocardiogram; ED: emergency department

Potassium was found to be 2.7 so IV potassium, magnesium, bicarbonate, and saline were started with lidocaine chosen as an antiarrhythmic (with which cardiology and poison control had agreed). The remaining basic labs, as well as toxicology and cardiac enzymes, were normal. Lorazepam was also used to control agitation. The electrocardiogram (ECG) showed tachycardia, which was different from the patient’s baseline atrial-paced rhythm. This ECG (Figure 1) was obtained when the patient was not in polymorphic ventricular tachycardia, which was only shown on the monitor for several seconds before being detected and electrically cardioverted by the AICD. Chest X-ray was normal. After treatment in the ED and intensive care unit (ICU), the shocks eventually became less frequent as electrolytes normalized. The patient was eventually removed from the lidocaine drip and discharged home five days later with the normalization of ECG over time (Figures 2-4).

## Discussion

Loperamide overdose is becoming a growing concern in the field of emergency medicine, secondary to the opioid epidemic. The over-the-counter anti-diarrheal medication is intended to only utilize mu-opioid receptors in the myenteric plexus of the gastrointestinal (GI) tract to slow intestinal motility. Unfortunately, in an overdose, specificity for the gastrointestinal tract is overwhelmed and the drug, including its opioid effects, becomes systemic [5]. Patients use high-dose loperamide for symptoms of opioid withdrawal and even attempt to recreationally overdose on this medication with doses of the medication over 100 times the recommended 16 mg daily maximum. Patients with a predisposition to arrhythmia, such as long QT syndrome in this case, are especially at risk due to the action of loperamide on potassium channels, resulting in QT prolongation, and on sodium channels, resulting in QRS prolongation [4]. Unfortunately, the number of cases of loperamide overdose continues to rise by as high as 91% from 2010-2015 [6], highlighting the increasing trend of the overdose on this medication [2,7-8]. Many cases have been reported showing arrhythmia in patients who overdose on loperamide to the point where the Food and Drug Administration (FDA) issued a warning in June 2016 about the potential cardiotoxic side effects of loperamide in an attempt to dissuade potential abusers [3]. Although acute overdose to achieve euphoria is common, patients also suffer from chronic overdoses because they use daily high-doses to control opioid withdrawal symptoms in place of drugs like methadone [9]. The overdose of loperamide can also be seen in individuals with chronic diarrhea who take doses that are much too high [10].

In this case, the patient had been a chronic abuser of loperamide and had been diagnosed by his cardiologist with long QT syndrome for which an AICD had been placed. Despite having been admitted to the ICU multiple times for acute loperamide overdose, he overdosed again.

Highlights in the management of this patient included cardioversion for hemodynamic instability and prompt correction of electrolyte deficiencies. The AICD handled electrical cardioversion, so our attention was turned to correcting electrolyte deficiencies.

The use of magnesium, calcium, potassium, bicarbonate, antiarrhythmics (lidocaine), naloxone, and activated charcoal should be considered in cases of loperamide overdose [11]. We used IV magnesium due to the patient’s history of long QT syndrome and frequent runs of polymorphic wide-complex tachycardia. IV potassium was given to replace the low potassium of 2.7. IV sodium bicarbonate was used for the wide-complex nature of the patient’s tachycardia. Lidocaine was utilized to prevent runs of wide complex tachycardia. Naloxone was considered in this patient, but he was in agitated delirium so we could not give it. We also did not give charcoal because of the delirium and possibility of the loss of airway reflexes. Rather than the usual one-hour post-ingestion, charcoal can be given up to two to four hours after the ingestion of loperamide because of the diminished intestinal transit that results from the actions of loperamide on the myenteric plexus [12].

## Conclusions

The number of cases of loperamide overdose called to poison control centers is increasing, as this drug becomes a popular way to achieve euphoria or fend off opioid withdrawal. Due to the cardiac implications of overdosing on this medication, the emergency physician has no choice but to be aware of the presence of this entity. Being aware of the common electrolyte disturbances and arrhythmias associated with a loperamide overdose can help identify and treat this condition in an acute toxicology patient.
